# Transcriptional Reprogramming of Cancer Metabolism: *Tricholoma terreum* Inhibits Nucleotide Biosynthesis and Energy Flux in MCF-7 Cells by Downregulating *DHFR*, *TK1*, and *ENO1*

**DOI:** 10.3390/ijms27083626

**Published:** 2026-04-18

**Authors:** Levent Gülüm, Emrah Güler, Emir Çapkınoğlu, Ayşe Büşranur Çelik, Yusuf Tutar

**Affiliations:** 1 Department of Plant and Animal Production, Mudurnu Süreyya Astarcı Vocational College, Bolu Abant İzzet Baysal University, Bolu 14030, Türkiye; leventgulum@ibu.edu.tr; 2 Medicinal and Aromatic Plants Research Group, Innovative Food Technologies Development Application and Research Center, Bolu Abant Izzet Baysal University, Bolu 14030, Türkiye; 3 Department of Horticulture, Faculty of Agriculture, Bolu Abant İzzet Baysal University, Bolu 14030, Türkiye; emrahguler6@gmail.com; 4 Horticulture and Post-Harvest Research Group, Innovative Food Technologies Development Application and Research Center, Bolu Abant Izzet Baysal University, Bolu 14030, Türkiye; 5 Department of General Surgery, School of Medicine, Acibadem Mehmet Ali Aydinlar University, Istanbul 34638, Türkiye; 6 Faculty of Medicine, Department of Basic Medical Sciences, Division of Biochemistry, Recep Tayyip Erdogan University, Rize 53020, Türkiye; ayseclk1899@gmail.com (A.B.Ç.); yusuf.tutar@erdogan.edu.tr (Y.T.); 7 Molecular Oncology Program, Health Sciences Institute, Recep Tayyip Erdogan University, Rize 53100, Türkiye; 8 Molecular Medicine Program, Health Sciences Institute, Recep Tayyip Erdogan University, Rize 53100, Türkiye; 9 Rize Training and Research Hospital, Recep Tayyip Erdogan University, Rize 53020, Türkiye; 10 Department of Basic Pharmaceutical Sciences, Division of Biochemistry, Faculty of Pharmacy, University of Health Sciences, Istanbul 34668, Türkiye

**Keywords:** *Tricholoma terreum*, MCF-7 cells, metabolic reprogramming, *SLC7A11*, apoptosis, volatile organic compounds (VOCs)

## Abstract

*Tricholoma terreum*, a mushroom rich in bioactive compounds, exhibits notable antioxidant and anticancer properties. Despite its traditional use, its effects on breast cancer metabolism remain underexplored. Here, we conducted comprehensive phytochemical and volatile organic compound profiling of *T. terreum* extracts and evaluated their cytotoxicity against MCF-7 breast cancer cells. Using SPME–GC–MS and HPLC, we identified a complex chemical matrix dominated by organic acids (acetic acid, 43.85%) and nitrogen-containing heterocyclics (2-acetylpyridine, 15.19%), alongside significant phenolic acids such as gallic acid and syringic acid. Biological assays indicated that the ethanol extract showed notable cytotoxic effects, reducing MCF-7 cell viability to 3.64% after 72 h, while higher viability was preserved in healthy CCD-1072sk fibroblast cells. Using cell viability assays, flow cytometry, and gene expression analysis, we found that ethanol extracts selectively reduced cancer cell viability, induced G0/G1 cell cycle arrest (71.92%), and promoted apoptosis. Mechanistically, treatment downregulated key nucleotide biosynthesis genes (*DHFR*, *TK1*) and the glycolytic enzyme gene (*ENO1*), while upregulating the oxidative stress response gene *SLC7A11* (18.32-fold), suggesting disruption of cancer metabolic pathways. These findings reveal a metabolic reprogramming effect of *T. terreum* extracts, highlighting their potential as metabolism-targeted agents in breast cancer therapy. Further studies are warranted to validate these effects in vivo and isolate active constituents.

## 1. Introduction

*T. terreum*, commonly known as the Grey Knight, is a widely consumed wild mushroom in temperate regions; while many ethnomycological and mycological sources classify it as edible and traditionally used as a culinary item in Europe, there is ongoing debate in the literature about potential toxicity, with some studies reporting concerns about toxic metabolites and contrasting regulatory judgments [1,2,3,4,5]. *T. terreum* is a reservoir of high-value bioactive compounds, primarily polysaccharides (beta-glucans) and phenolic compounds [6]. These constituents form the bedrock of the mushroom’s nutritional and medicinal value. Polysaccharides, particularly those characterized by unique branching structures, serve as potent biological response modifiers, while phenolics provide a robust secondary metabolite profile [7]. Recent studies suggest that these two groups work synergistically, enhancing the mushroom’s overall health-promoting properties beyond what individual components could achieve alone [8]. This complex chemical matrix is further enriched by highly oxygenated meroterpenoids and specific fatty acids, which have begun to draw significant attention for their specialized biological activities [9,10].

The therapeutic hallmark of *T. terreum* lies in its superior antioxidative capacity, which plays a critical role in mitigating oxidative stress, a known precursor to chronic diseases and DNA damage [11]. The mushroom’s extracts demonstrate high efficiency in scavenging free radicals and inhibiting enzyme activities that lead to cellular oxidation [12,13]. This protection is largely attributed to its richness in flavonoids and phenolic acids, which help maintain cellular homeostasis [14]. By neutralizing reactive oxygen species (ROS), *T. terreum* acts as a natural shield, potentially reducing the risk of inflammatory conditions and various malignancies associated with long-term oxidative damage [8,15].

Beyond general protection, *T. terreum* exhibits targeted cytotoxic effects against various cancer cell lines, including breast and liver cancers [16,17]. Cancer cells typically undergo metabolic reprogramming, such as the Warburg effect, to fuel rapid proliferation [18,19]. Research indicates that the bioactive terpenoids and polysaccharides in *T. terreum* can disrupt these metabolic dependencies, specifically targeting pathways like *PI3K*/*AKT*/*mTOR* and *MAPK* [20,21]. By inhibiting key glycolytic enzymes and modulating the expression of oncogenes like *KRAS*, these compounds can induce apoptosis (programmed cell death) and suppress tumor growth, offering a multi-faceted attack on the metabolic “addictions” of malignant cells [22,23].

The integration of traditional mushroom use with modern pharmacological analysis positions *T. terreum* as a prime candidate for chemopreventive agent development [24]. Its ability to exert cytotoxic effects comparable to established drugs like cisplatin, while providing anti-inflammatory and immune-enhancing benefits, highlights its versatility [9,25]. However, to transition from laboratory findings to clinical dietary strategies, further research using advanced profiling like GC-MS is essential to isolate specific active metabolites [26,27]. Elucidating how these aromatic compounds interact with human cellular signaling pathways is essential for determining the potential of *T. terreum* as a functional food in cancer and chronic disease prevention [8,28].

In this study, we evaluate the cytotoxic and pro-apoptotic effects of ethanol and methanol extracts from *T. terreum* on the MCF-7 human breast cancer cell line. We hypothesize that unique aromatic compounds and meroterpenoids present in *T. terreum* can disrupt metabolic pathways critical for tumor growth and survival in breast cancer cells. By investigating molecular mechanisms such as apoptosis induction and inhibition of metabolic proliferation, we aim to identify bioactive compounds with potential as novel therapeutic agents or dietary supplements for adjuvant breast cancer therapy. Our findings are intended to bridge traditional ethnomycological knowledge with targeted oncological applications.

## 2. Results

### 2.1. Total Bioactive Constituents

#### Total Phenolics, Flavonoids, and Anthocyanins

The phytochemical analysis of *T*. *terreum* revealed distinct profiles depending on the extraction solvent used. While the methanol extract yielded a significantly higher total phenolic content (TPC) at 1.08 mg/g GAE (gallic acid equivalent) compared to the ethanol extract’s 0.65 mg/g GAE, the trend reversed for flavonoid concentrations. Specifically, ethanol proved more efficient for isolating flavonoids, achieving a TFC value of 0.62 mg/g QE (quercetin equivalent), more than double that of the 0.28 mg/g QE found in its methanol counterpart. A similar difference was observed in the anthocyanin levels; the ethanol extract was notably richer in these pigments, with a value of 0.61 mg/g ME (malvidin-3-glucoside equivalent), whereas the methanol extract reached a lower concentration of 0.46 mg/g ME (Table 1). These results suggest that while methanol is the superior solvent for general phenolics in *T*. *terreum*, ethanol is more effective for capturing specific antioxidant sub-classes such as flavonoids and anthocyanins.

### 2.2. Macronutrient Profile of T. terreum

#### Soluble Proteins and Total Carbohydrates

Nutritional profiling of the extracts highlighted significant differences in yield based on solvent polarity. The methanol extract proved superior for protein recovery, reaching a concentration of 18.64%, while the ethanol extract yielded a lower value of 13.15%. Conversely, the carbohydrate content showed a different distribution; the ethanol extract was notably richer in sugars and fibers with a concentration of 60.06%, whereas the methanol extract contained 53.77% (Table 2). These findings indicate that while methanol is more effective for isolating the nitrogenous components of *T. terreum*, ethanol remains the more efficient choice for capturing its carbohydrate-rich matrix.

### 2.3. Antioxidant Capacity of T. terreum Extracts

#### DPPH, ABTS, FRAP, and CUPRAC Activities in *T. terreum*

The antioxidant capacity of *T. terreum* varied significantly across the four assays, highlighting how different solvents isolate specific radical-scavenging compounds. In the DPPH assay, the methanol extract demonstrated over twice the potency of the ethanol extract, reaching 1.04 mg/g compared to 0.49 mg/g. An even more dramatic disparity was observed in the CUPRAC results, where methanol proved exceptionally effective with a value of 10.30 mg/g, significantly exceeding the 1.96 mg/g recorded for ethanol. The ABTS assay further confirmed the superior efficiency of the methanol extract, which yielded 3.19 mg/g, while the ethanol extract reached 1.72 mg/g. However, a notable shift in the trend was observed in the FRAP assay, where the ethanol extract exhibited superior reducing power at 3.00 mg/g, surpassing the 2.06 mg/g found in the methanol extract (Table 3).

### 2.4. Targeted Phytochemicals in T. terreum

#### HPLC-Based Phenolic Profile of *T. terreum* Extracts

The mushroom phenolic and organic acid profiles of *T. terreum* revealed notable quantitative differences between ethanol and methanol extracts. In general, the methanol extract exhibited higher total phenolic and organic acid contents compared to the ethanol extract. Among phenolic acids, gallic acid was detected at a remarkably high level in the methanol extract (725.59 µg/g), whereas it was present at a considerably lower concentration in the ethanol extract (12.92 µg/g). Similarly, 4-aminobenzoic acid and vanillic acid were detected exclusively in the methanol extract at concentrations of 117.16 µg/g and 237.25 µg/g, respectively. Syringic acid was one of the dominant compounds in both extract types, reaching a higher concentration in the methanol extract (567.90 µg/g) compared to the ethanol extract (375.09 µg/g). Regarding flavonoid compounds, pro catechin and chlorogenic acid were detected in both extract types. Catechin was higher in the ethanol extract (106.03 µg/g) than in the methanol extract (95.73 µg/g). Chlorogenic acid also showed slightly higher levels in the ethanol extract (60.80 µg/g) compared to the methanol extract (47.01 µg/g). Quercitrin was another major flavonoid compound, with concentrations of 277.72 µg/g in the ethanol extract and 209.08 µg/g in the methanol extract. Analysis of catechin derivatives showed that (−)-epicatechin was substantially higher in the ethanol extract (737.95 µg/g) than in the methanol extract (124.56 µg/g), whereas (+)-catechin was not detected in either extract. In terms of hydroxybenzoic and hydroxycinnamic acid derivatives, ferulic acid was measured at 63.19 µg/g in the ethanol extract and 101.14 µg/g in the methanol extract. Synapic acid was detected only in the methanol extract (62.47 µg/g), while coumaric acid and rutin trihydrate were not detected in either extract. For the organic acid profile, succinic acid was higher in the methanol extract (247.58 µg/g) compared to the ethanol extract (134.43 µg/g). Salicylic acid was not detected in either extract type (Table 4).

### 2.5. Volatile Metabolites of T. terrum

#### SPME–GC–MS-Based Aroma Characteristics in *T. terreum*

Chemical profiling of *T. terreum* via SPME–GC–MS identified a diverse array of volatile organic compounds (VOCs), with organic acids emerging as the most dominant group, accounting for 45.12% of the total area. This fraction was primarily driven by acetic acid, which alone represented 43.85% of the profile, followed by a smaller contribution of hexanoic acid at 1.27%. Nitrogen-containing heterocyclic compounds constituted the second most prevalent group at 22.60%. This category was remarkably high in 2-acetylpyridine (15.19%), alongside several pyrazine derivatives such as 2,6-dimethylpyrazine and 2,3,5-trimethylpyrazine, which are often associated with the characteristic earthy and nutty aromas of mushrooms. Other significant chemical classes included ketones (7.15%), led by 2-octanone at 4.01%, and alcohols (5.95%), with tridecanol being the most prominent at 2.33%. Alkanes and alkenes comprised 4.62% of the area, featuring compounds like 1-nonadecene. Meanwhile, aldehydes like hexanal and benzaldehyde contributed 3.49% to the total profile. More specialized groups such as lactones (2.95%), esters (1.26%), and phenolic compounds like o-cresol (1.07%) were also identified (Table 5). The presence of terpenoid derivatives and aromatic vinyls like dimethylstyrene, though in lower percentages, adds to the complex chemical matrix that likely contributes to the mushroom’s overall biological and therapeutic potential.

### 2.6. Cell-Based Bioactivities of T. terreum Exracts

**Figure 1:**
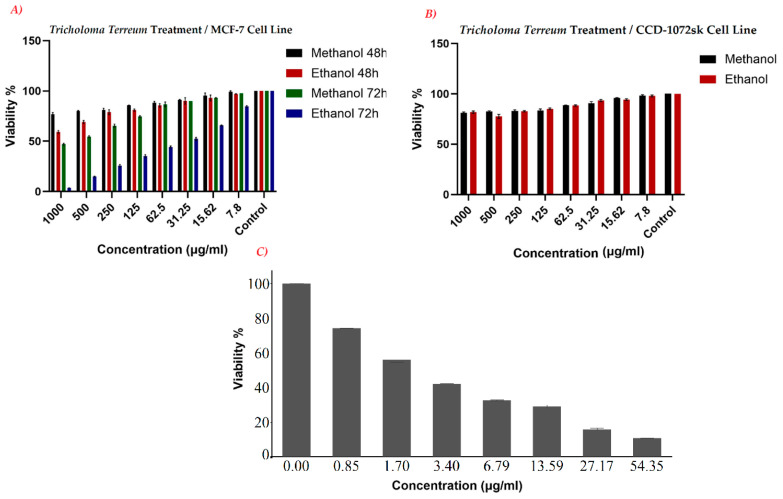
Cell viabilities induced by *T. terreum* extract treatments in MCF-7 breast cancer cells (**A**) and healthy cells (**B**), and by the clinical medicine (doxorubicin) in MCF-7 cells (**C**).

The study demonstrated that *T. terreum* extracts caused a significant, time- and dose-dependent decrease in the viability of MCF-7 breast cancer cells (ATCC no: HTB-22). After 48 h, the ethanol extract reduced cell viability to 59.34% at the highest concentration tested (1000 µg/mL), while the methanol extract resulted in 76.68% viability. When the exposure time was extended to 72 h, the cytotoxic effects became more pronounced: the ethanol extract at 1000 µg/mL reduced cell viability to 3.64%, and even at 31.25 µg/mL, viability was reduced to 52.42% (IC_50_ = 50.96 µg/mL). The methanol extract at 72 h achieved a viability of 47.17% at 1000 µg/mL, with an IC_50_ of 783.13 µg/mL (Figure 1A).

An important aspect of this research was to assess whether the extracts selectively target malignant cells while sparing healthy tissue. When tested on healthy human skin fibroblasts (CCD-1072sk, ATCC no: CRL-2088), both methanol and ethanol extracts maintained high cell viability, with values of 81.10% and 81.96%, respectively, at the highest concentration tested (1000 µg/mL). At concentrations below 125 µg/mL, viability remained above 85%, indicating selective cytotoxicity toward cancer cells (Figure 1B). The selectivity index (SI) was approximately 1.28 for methanol and greater than 24.28 for the ethanol extract, suggesting that the ethanol-extracted bioactive compounds in *T. terreum* preferentially affect cancer cells over normal cells.

The efficacy of *T. terreum* extracts was further assessed by comparison with Doxorubicin, a standard chemotherapeutic agent used as a positive control. Doxorubicin exhibited a potent IC_50_ of 3.46 µg/mL, reducing MCF-7 cell viability to 10.85% at a concentration of 54.35 µg/mL. Notably, the ethanol extract of *T. terreum* at 72 h reduced cell viability to 3.64% at 1000 µg/mL (Figure 1C). These results support the potential of the aromatic and phenolic constituents of *T. terreum* as promising candidates for the development of new, naturally derived cancer therapies with selective cytotoxicity toward cancer cells.

### 2.7. Flow Cytometry-Based Cellular Responses to T. terreum Treatment

#### 2.7.1. Changes in Cell Cycle

**Figure 2:**
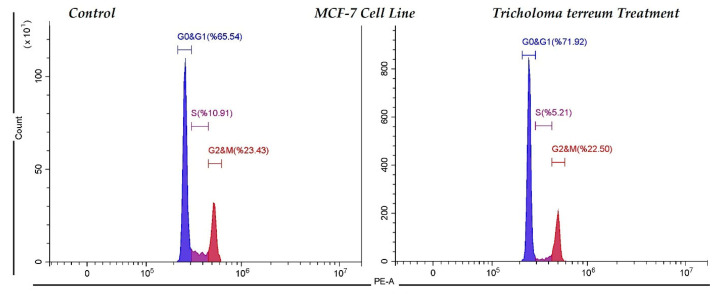
Cell cycle induction in control (untreated) and *T. terreum* ethanol extract-treated MCF-7 breast cancer cells.

Cell cycle distribution of MCF-7 cells following *T. terreum* treatment was evaluated by flow cytometry and compared with the untreated control group (Figure 2). In the control group, cells were predominantly distributed in the G0/G1 phase (65.54%), followed by G2/M (23.43%) and S phase (10.91%). Upon treatment with *T. terreum*, a marked increase in the G0/G1 population was observed (71.92%), accompanied by a reduction in the S phase fraction (5.21%). The G2/M phase showed a slight decrease to 22.50% compared to the control group. These findings indicate that *T. terreum* treatment induces cell cycle arrest predominantly at the G0/G1 phase in MCF-7 cells, thereby suppressing DNA synthesis as reflected by the reduced S phase population. The accumulation of cells in G0/G1 suggests a potential antiproliferative effect mediated through inhibition of cell cycle progression.

#### 2.7.2. Apoptosis Induced by *T. terreum*

**Figure 3:**
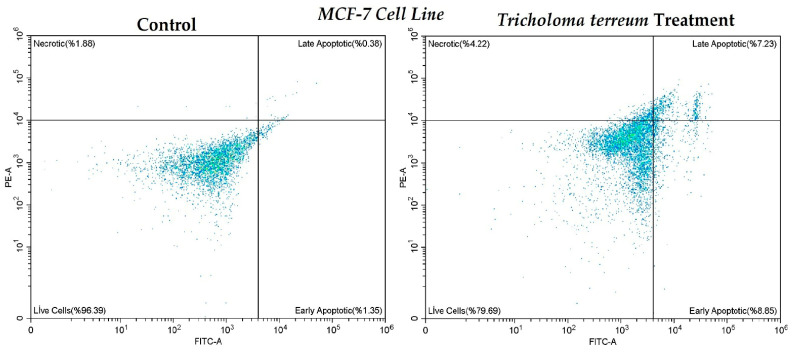
The induction of apoptosis in untreated and *T. terreum* ethanol extract-treated MCF-7 breast cancer cells.

Apoptotic cell death in MCF-7 cells following *T. terreum* treatment was evaluated using Annexin V–FITC/PI staining and flow cytometric analysis (Figure 3). In the control group, the majority of cells were viable (96.39%), with low percentages of early apoptotic (1.35%), late apoptotic (0.38%), and necrotic cells (1.88%), indicating normal cell viability under untreated conditions. In contrast, treatment with *T. terreum* markedly altered the apoptotic profile of MCF-7 cells. The proportion of viable cells decreased to 79.69%, while early apoptotic and late apoptotic populations increased to 8.85% and 7.23%, respectively. Additionally, the necrotic cell population rose to 4.22%. These findings suggest that *T. terreum* treatment significantly induces apoptosis in MCF-7 cells, as evidenced by the substantial increase in both early and late apoptotic fractions. The shift from viable to apoptotic cell populations supports the cytotoxic and pro-apoptotic potential of the treatment.

### 2.8. Gene Transciption in MCF-7 by T. terreum Treatment

#### RT-qPCR-Based Genetic Transcription and Reactome Results

Out of the 46 submitted genes, 44 were successfully mapped to the Reactome database, identifying a total of 255 distinct pathways. The analysis identified “Metabolism” as the most significantly enriched top-level category, with a highly significant *p*-value of 1.11 × 10^−16^. This indicates that the primary biological impact of the treatment is a comprehensive restructuring of the cell’s energetic and biosynthetic priorities.

One of the most prominent shifts occurs within central carbon metabolism, specifically affecting glucose and pentose phosphate pathways. The analysis shows a pronounced activation of “Glucose Metabolism” and “Glycolysis”, supported by the significant upregulation of *HK2* (6.39-fold), *PFKFB4* (3.33-fold), *SLC2A1* (1.81-fold), and *SLC2A2* (5.22-fold). Conversely, several downstream glycolytic enzymes were downregulated, including *ENO1* (−3.64-fold), *TPI1* (−2.17-fold), and *PGAM1* (−1.42-fold). Notably, the “Pentose Phosphate Pathway” is strongly represented through the massive induction of *G6PD* (12.25-fold), which highlights an increased capacity for NADPH production and oxidative stress adaptation.

The treatment also induces a strategic remodeling of mitochondrial and lipid metabolism. Pathways related to the “Citric Acid Cycle (TCA cycle)” and “Aerobic Respiration” were significantly enriched but showed a general trend of suppression. Key mitochondrial genes such as *OGDH* (−1.43-fold), *SDHA* (−1.59-fold), *FH* (−1.80-fold), and *IDH1* (−1.74-fold) were downregulated, suggesting an attenuation of oxidative phosphorylation. Simultaneously, lipid metabolism pathways like “Fatty Acyl-CoA Biosynthesis” and “Regulation of lipid metabolism by PPARalpha” were significantly modulated. This is characterized by the upregulation of *HMGCS1* (3.13-fold), *ACLY* (1.63-fold), *ACSL1* (1.91-fold), and *ACSL3* (3.35-fold), while ketone body utilization was suppressed through the marked downregulation of *ACSS3* (−13.3-fold) and *ACAT1* (−10.8-fold).

**Figure 4:**
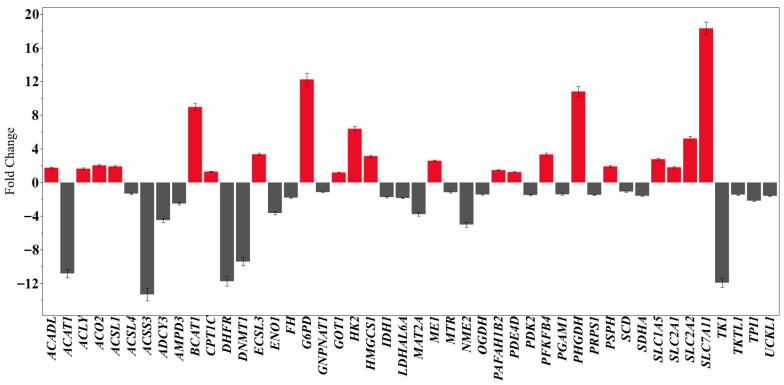
Effects of *T. terreum* extracts on the MCF-7 breast cancer cell gene expression profile.

Furthermore, the Reactome report highlights a major inhibition of pathways required for cell proliferation and genetic maintenance. The “G1/S-Specific Transcription” pathway was significantly enriched and characterized by the severe downregulation of *DHFR* (−11.8-fold) and *TK1* (−11.9-fold), which are essential for nucleotide biosynthesis and DNA replication. This suppression is further evidenced by the downregulation of *DNMT1* (−9.42-fold) and *NME2* (−5.01-fold), pointing to a diminished capacity for DNA methylation and nucleotide metabolism. In parallel, amino acid transport and redox buffering pathways were enriched through the strong upregulation of *SLC7A11* (18.32-fold) and *BCAT1* (8.97-fold), suggesting a shift toward cellular survival and stress response over division (Figure 4).

## 3. Discussion

The protein and carbohydrate contents of *T. terreum* were significantly influenced by the choice of extraction solvent, revealing distinct nutritional profiles. The protein content was found to be higher in the methanol extract (18.64%) compared to the ethanol extract (13.15%). This finding for the methanol extract is highly compatible with the results reported by Barros et al. (2007) [29] for *Tricoloma portentosum* (16.20%) and other wild Portuguese mushrooms. Several studies report protein contents and emphasize that macronutrient profiles, including protein, vary with extraction method and solvent, which in turn affects the measured carbohydrate-to-protein balance in mushroom samples [29]. Turfan et al. report that protein and carbohydrate contents vary among wild-growing and cultivated mushrooms, with nutrient composition significantly influenced by species and environmental factors; though not always framed in solvent extraction terms, they illustrate that proximate composition (including soluble protein) is variable across species and conditions [30]. A broader metabolomic/nutritional profiling across *Tricholoma* species, including *T. terreum*, indicates substantial variation in carbohydrate contents among species and samples, with protein content showing comparable variability. For *T. terreum*, carbohydrate contents ranged widely in different studies, reflecting species-level differences and methodological variation, which would include extraction choices in analytical workflows like solvent systems used for extraction or hydrolysis if measuring soluble vs. total protein [12,30,31]. This supports the claim that the reported protein content can be solvent-dependent in practice when different extraction/separation steps are used.

Solvent choice significantly dictated the recovery of bioactive compounds. While we found the methanol extract to yield the highest TPC, the ethanol extract was remarkably higher in flavonoids and anthocyanins. In contrast, previous studies on various mushroom species, including *T. terreum*, often highlight methanol as the universal solvent for total phenolics [32]. The significant difference in flavonoid recovery in our study may be due to the specific flavonoid glycosides in *T. terreum* that may have a higher affinity for the 80% ethanol-water system compared to the more traditional methanolic extractions reported by Jacinto-Azevedo et al. [33]. Furthermore, our DPPH and CUPRAC results for the methanol extract indicate a higher general radical scavenging capacity and antioxidant power, which aligns with findings by Sevindik et al. [34] regarding the robust antioxidant potential of *Tricholoma* species.

Phenolic acids and flavonoids identified in *Tricholoma* spp., such as chlorogenic acid and caffeoylquinic acid, frequently appear as dominant phenolic constituents across several mushroom genera and are highlighted as key contributors to antioxidant activity [35,36]. Within the *Tricholoma* genus, these hydroxycinnamic acids are recurrently noted as prominent in species profiles, specifically in *T. scalpturatum*, and are fundamentally associated with the antioxidant capacity of mushroom extracts [35]. While explicit quantification for species like *T. terreum* or *T. fracticum* is not always available in candidate summaries, the emphasis on chlorogenic acid as a principal phenolic is consistently reported across multiple genera and remains highly relevant to *Tricholoma*-containing matrices [35]. Furthermore, protocatechuic, vanillic, gallic, caffeic, and ferulic acids are repeatedly identified as major phenolic acids with significant antioxidative roles, as noted in various surveys where these compounds dominate the profiles of edible mushrooms [36,37,38]. Collectively, previous research implies that *Tricholoma* spp. likely possess a comparable suite of phenolic acids, even if individual species exhibit variable abundances depending on the solvent and sample type used. In this study, phenolic acids such as gallic, syringic, and vanillic acids were found to be abundant in the methanol extract of *T. terreum*. Contrary to existing literature, chlorogenic acid was not the predominant phenolic compound; instead, gallic acid was the primary phenolic substance in the methanol extract, while (-)-epicatechin was the predominant compound in the ethanol extract. The findings regarding individual phenolics are consistent with the TPC and TFC results, which indicated that the methanol extract had a high TPC, whereas the ethanol extract was rich in TFC.

VOCs are fundamental to the aroma, flavor, and ecological identity of the *Tricholoma* genus, serving as critical markers for species differentiation, authentication, and quality control. Consistent with the literature, including the comparative studies by Taşkın et al. [39], which highlighted the richness of volatile acids and alcohols in species like *T. anatolicum*, the current data reveals an overwhelming dominance of organic acids (45.12%), led primarily by acetic acid. Furthermore, the significant presence of nitrogen-containing heterocyclic compounds (22.60%), including various pyrazines and 2-acetylpyridine, strongly corroborates the established role of these metabolites as key odor contributors and potential chemotaxonomic markers within the genus [3,40]. While the literature on focal species like *T. matsutake* often emphasizes C8 compounds, specifically 1-octen-3-ol, as the primary mushroom odorant [41,42], the absence of this specific alcohol in the present profile, substituted by 2-octanone (4.01%) and hexanal (1.99%), suggests notable variations likely driven by species-specific genetics or post-harvest factors. As noted by Qin et al. (2025) and Zhao et al. (2024), VOC bouquets are highly sensitive to tissue maturity, storage, and processing conditions like drying, which can significantly alter the concentration of C8 volatiles [43,44]. Ultimately, the observed chemical profile, characterized by a high proportion of acids, nitrogenous heterocycles, and ketones, mirrors the complex and taxon-specific volatile diversity of *Tricholoma* spp., reinforcing the necessity for standardized analytical protocols to accurately interpret these fungal fingerprints.

The anticancer potential of VOCs from *Tricholoma* species is supported by a growing body of evidence linking mushroom-derived volatile extracts to cytotoxic and immunomodulatory activities [45]. The high concentration of acetic acid (43.85%) and 2-acetylpyridine (15.19%) identified in the volatile profile of *T. terreum* supports the emerging role of VOCs as bioactive markers in edible fungi [45]. This aligns with recent transcriptomic evidence suggesting that dietary polyphenols exert significant regulatory control over metabolic gene expression [46]. In this context, the high anticancer potential demonstrated by the *T. terreum* extract may be due to its rich profile of VOCs and phenolics; specifically, the detection of o-cresol and various phenolic acids like gallic acid and syringic acid reinforces the multi-targeted cytotoxicity model, where diverse metabolites work synergistically to disrupt cellular homeostasis [14,47]. While the presence of bioactive chemical families, particularly indole-type volatiles and C8 aliphatics, provides a strong mechanistic basis for investigating cytotoxic effects [48,49], methodological variability and the lack of absolute quantification for many taxa pose challenges for causal attribution. Furthermore, as we also did not study the specific activity of VOCs and other individual compounds, this issue should be considered a potential research area. Consequently, future research must prioritize bioassay-guided fractionation, standardized sampling, and rigorous taxonomic verification to move beyond broad extract-based observations and validate the specific anticancer efficacy of individual *Tricholoma* volatiles within standardized models [39,50].

Mushroom-derived extracts exhibit a multi-faceted anticancer profile characterized by direct cytotoxic effects, potent immunomodulation, and anti-metastatic activities, driven by a diverse array of bioactive compounds such as beta-glucans, terpenoids, and polyphenols [51,52]. The reduction in MCF-7 cell viability to 3.64% (ethanol, 72 h) observed in our research represents a noteworthy finding in natural product pharmacology, and it compares favorably with recent reports on other natural extracts such as *Corylus avellana* [53]. At its highest dose (1000 µg/mL), the ethanol extract yielded results that were competitive with lower doses of the clinical drug doxorubicin (10.85% viability) under identical conditions [54]. This potential may be attributed to the mushroom’s rich chemical fingerprint; specifically, the high concentrations of acetic acid (43.85%) and 2-acetylpyridine (15.19%) identified in the volatile profile of *T. terreum* support the emerging role of VOCs as bioactive markers [45]. This aligns with recent transcriptomic evidence suggesting that dietary polyphenols, including the o-cresol, gallic acid, and syringic acid detected in our samples, exert significant regulatory control over metabolic gene expression and may work synergistically to disrupt cellular homeostasis [14,46,47].

Mechanistically, our flow cytometric data indicate that *T. terreum* exerts an antiproliferative effect by increasing the proportion of MCF-7 cells in the G0/G1 phase (71.92%), similar to effects observed with certain cell cycle inhibitors [55]. Our study further suggests a deeper genetic explanation through the observed downregulation of *DHFR* (−11.8 fold) and *TK1* (−11.9 fold), enzymes required for nucleotide biosynthesis [56]. By suppressing these key pathways, the mushroom extract may hinder processes involved in DNA replication, which is associated with the observed increase in apoptosis to 16.08% in our treated groups. Importantly, our extracts maintained over 81% viability in healthy human skin fibroblasts (CCD-1072sk), suggesting a degree of selective toxicity toward malignant cells [57].

A significant observation in this research involves the apparent manipulation of metabolic reprogramming, a concept also explored in recent studies of *G. lucidum* [32]. While cancer cells typically rely on accelerated glycolysis to fuel proliferation [58,59], our results suggest a “metabolic trap.” Despite the upregulation of glucose transporters (*SLC2A1*/*2*), the simultaneous inhibition of downstream glycolytic enzymes *ENO1* (−3.64 fold) and *TPI1* (−2.17 fold) may prevent the cell from efficiently converting imported glucose into usable energy [60,61].

**Figure 5:**
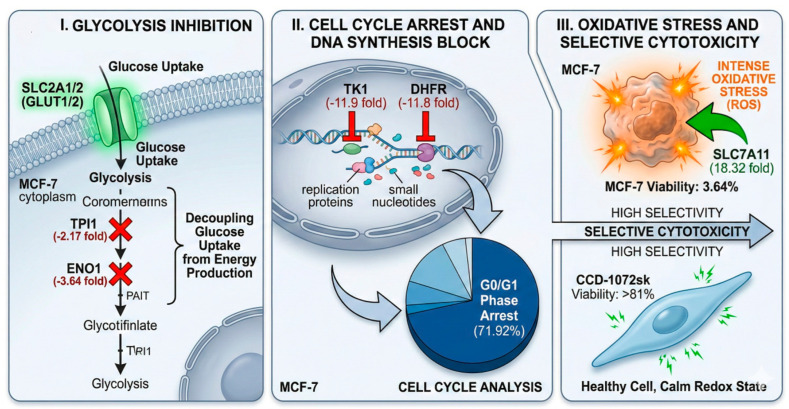
A hypothetical schematic view of key findings obtained in this study on MCF-7 breast cancer cells by induction of *T. terreum* ethanol extract. The figure was generated by Nano Banana 2.

This metabolic bottleneck, coupled with the substantial 18.32-fold upregulation of *SLC7A11*, suggests that the cells may have shifted their energetic priority toward a survival-based oxidative stress response [62]. This potential decoupling of nutrient uptake from energy production may reduce the availability of biosynthetic precursors needed for cell division, as described in systemic models of metabolic crisis in malignant tissues [63,64]. A mechanistic view of these discussed metabolic effects of *T. terreum* is provided in Figure 5. However, it is important to note that this study was conducted on in vitro cells, and the mechanistic data provided are derived primarily from gene expression analysis. Consequently, these results provide hypothetical outcomes that necessitate further validation to establish the exact biological mechanism. To confirm these findings beyond the transcriptomic level, protein-level validations and further studies at the animal or human level are required.

## 4. Materials and Methods

### 4.1. Biological Material and Extract Preparation

#### Mushroom Source, Cultivation Conditions, and Extraction Approach

Fresh fruiting bodies of *T. terreum* were obtained from a local market in Türkiye during the natural harvesting season. The identification was provided by Prof. Dr. Fuat Bozok. A voucher number, L. Gülüm-1002, was assigned to this material and is now preserved in the biochemistry laboratory of the Horticulture Department at Bolu Abant İzzet Baysal University. The dried and powdered mushroom material was extracted using aqueous organic solvents, specifically 80% ethanol and 80% methanol, following a previously described protocol with minor modifications [65]. In brief, the samples were incubated at room temperature and subjected to sonication to enhance extraction efficiency. After sonication, the mixture was filtered and then centrifuged. The supernatants were concentrated under reduced pressure and stored at −20 °C. For subsequent analyses, the extracts were dissolved in dimethyl sulfoxide (DMSO) to prepare stock solutions.

### 4.2. Chemical Composition and Antioxidant Characterization

#### 4.2.1. Quantification of Total Bioactive Constituents

##### Total Phenolics, Flavonoids, and Anthocyanins Assays

The TPC was measured using a microscale Folin–Ciocalteu-based spectrophotometric assay, which was adapted from previously published methods [66,67]. In this process, the extract was reacted with Folin–Ciocalteu reagent and sodium carbonate under controlled conditions. After incubating the mixture in the dark, the absorbance was recorded at 760 nm. Quantification was carried out using a gallic acid calibration curve, with the results expressed as GAE per gram of dry weight.

TFC was determined using a modified aluminum chloride-based colorimetric assay [65,68]. In this method, the extracts were sequentially reacted with sodium nitrite, aluminum chloride, and sodium hydroxide, and the absorbance was measured at 430 nm. Quantification was performed using a quercetin calibration curve, and the results were expressed as millimoles of quercetin equivalents per gram of dry weight.

Total anthocyanin content was assessed using an acidified ethanol method [69]. Samples were mixed with 30% ethanol containing 1% hydrochloric acid and analyzed spectrophotometrically at 540 nm. Anthocyanin levels were calculated as malvidin-3-glucoside equivalents per gram of dry weight using a specific equation.

Tant = 16.7 × A540 × Df

In this formula, Df represents the dilution factor.

#### 4.2.2. Macronutrient Profiling

##### Determination of Soluble Proteins and Total Carbohydrates

Soluble protein content was measured using a modified Bradford assay [70]. Quantification was performed using bovine serum albumin. In this assay, the sample reacted with Bradford reagent, and the absorbance was recorded at 595 nm. Bovine serum albumin was used to generate the standard calibration curve, and protein concentrations were calculated accordingly.

Total carbohydrate content was determined using a modified phenol-sulfuric acid method [71]. Samples were treated with phenol and concentrated sulfuric acid. Then, spectrophotometric measurement was performed using glucose as the reference standard. Carbohydrate levels were expressed as a percentage of the sample.

#### 4.2.3. Antioxidant Capacity Assessment

##### DPPH, ABTS, FRAP, and CUPRAC

The antioxidant capacity was evaluated using four complementary spectrophotometric assays, with results expressed as mg g TE.

DPPH radical scavenging activity was determined by reacting 100 µL of the extract with 1900 µL of an ethanolic DPPH solution, which was adjusted to an initial absorbance of 0.70–0.80 at 517 nm with a UV-Vis spectrophotometer (DLAB, SP-UV1000, Beijing, China). The mixture was then incubated for 15 min at room temperature, followed by measurement of the absorbance at the same wavelength [72]. DPPH scavenging activity was calculated against the Trolox standards that were prepared by the same procedure, and the exact activity in the samples was calculated using the equation obtained from the standards (R^2^ = 0.99). The results were expressed as mg/g TE.

CUPRAC activity was assessed through a reaction system containing 10 mM Cu^2+^, 7.5 mM neocuproine, and 1 M ammonium acetate buffer. The sample–reagent mixtures (100 µL:1900 µL) were incubated for 20 min at room temperature, and the absorbance was recorded at 450 nm with a UV-vis spectrophotometer (DLAB, SP-UV1100, China) [73]. The Trolox standards, serially diluted from 2 mg/mL, went through the identical procedure, and the results were calculated using the equation obtained from the standards (R^2^ = 0.99) and expressed as mg/g TE.

The ABTS+ scavenging capacity was measured using pre-formed ABTS radicals (7 mM ABTS with potassium persulfate), which were diluted to an absorbance of 0.70 ± 0.01 at 734 nm. After dilution, the extract was reacted for 15 min before measuring the absorbance at 734 nm [74]. A 1 mg/mL Trolox standard was serially diluted and used for the preparation of a standard curve. The ABTS scavenging activities of the samples were calculated using the equation obtained from the standard curve, and the results were expressed as mg/g TE.

FRAP was evaluated by mixing equal volumes of diluted reagent and the sample (100 µL each) to obtain a total reaction volume of 200 µL. The absorbance was measured after incubation under standard conditions at 593 nm using a microplate reader (Multiscan GO, Thermo Scientific, Waltham, MA, USA) [75]. The standards prepared by serially diluting 1 mg/mL Trolox went through the identical procedure and were read alongside the samples. The FRAP activities were calculated by using the equation obtained from Trolox standards (R^2^ = 0.99) and expressed as mg/g TE.

### 4.3. Targeted Phytochemical Profiling

#### HPLC-Based Phenolic Profiling

Phenolic profiling was performed using high-performance liquid chromatography (HPLC) following a protocol adapted from Rodriguez-Delgado et al. [76]. The analyses utilized a Waters HPLC system equipped with gradient pumps, an autosampler injector (20 µL loop), and UV–fluorescence detection. Chromatographic separation was achieved on a reversed-phase C18 column (150 × 3.9 mm, 4 µm), protected by a guard column. The elution process was conducted at a flow rate of 1.0 mL/min, employing a binary gradient consisting of solvent A (methanol/acetic acid/water, 10:2:88, *v*/*v*) and solvent B (methanol/acetic acid/water, 90:2:8, *v*/*v*). Detection occurred at 280 nm, while fluorescence signals were monitored with excitation/emission wavelengths of 278/360 nm and 330/374 nm. Peak homogeneity was confirmed through diode array spectral analysis, with acceptance criteria set at ≥99.5% spectral similarity. For quantitative determination, a broad range of phenolic standards was dissolved in methanol and serially diluted from an initial concentration of 80 mg/L. All standard and sample solutions were filtered through 0.45 µm cellulose acetate membranes be-fore analysis. An internal standard approach utilized 2,5-dihydroxybenzaldehyde (34.4 mg/L) to enhance analytical accuracy. Compound identification was based on matching retention times and spectral characteristics with reference standards, and calibration curves were generated using linear regression (R^2^ > 0.99). Final phenolic concentrations were calculated from their corresponding regression equations and expressed accordingly.

### 4.4. Volatile Metabolite Analysis

#### SPME–GC–MS-Based Aroma Characterization

Volatile metabolite analysis was conducted by modifying the SPME–GC–MS protocol described by Wang et al. [77]. The analyses were carried out using a Shimadzu GCMS-QP2010 system, which was equipped with an Rxi-5ms fused-silica capillary column (60 m × 0.25 mm i.d., 0.25 µm film thickness). In brief, approximately 0.9 g of finely ground and homogenized sample was placed in a 20 mL sealed headspace vial and equilibrated at 70 °C for 30 min. The headspace volatiles were then adsorbed using a 75 µm CAR/PDMS SPME fiber and thermally desorbed in the GC injector at 250 °C under splitless conditions. Chromatographic separation was achieved using helium as the carrier gas at a flow rate of 1.0 mL/min. A multi-step oven temperature program was followed, starting at 45 °C and gradually increasing to a final temperature of 240 °C, with intermediate holding and ramping stages to ensure efficient resolution of the volatile compounds. Mass spectrometric detection was performed in electron impact (EI) mode at 70 eV, scanning ions in the m/z range of 30–650. The temperatures of the interface, ion source, and quadrupole were maintained at 250 °C, 230 °C, and 150 °C, respectively. Volatile constituents were identified by matching mass spectra and calculated retention indices with those of reference standards and spectral libraries (FFNSC 1.2 and Wiley 7), using a C7–C20 n-alkane series for index calculation. The relative abundances of the volatile compounds were expressed as percentage peak area ratios, calculated by dividing the individual peak area by the total chromato-graphic area.

### 4.5. Cell-Based Bioactivity Assessment

#### Cell Culture Conditions

Cell-based assays were conducted by slightly modifying the protocol established by Gülüm et al. [67,78]. Human MCF-7 breast adenocarcinoma cells (ATCC) were maintained in Dulbecco’s Modified Eagle Medium (DMEM), supplemented with 10% fetal bovine serum and 1% glutamine. The cells were cultured at 37 °C in a humidified environment with 5% CO_2_. When the cells reached 80–90% confluence, they were subcultured using 0.25% trypsin–EDTA and plated in 96-well plates at densities ranging from 5 × 10^3^ to 1 × 10^4^ cells per well.

After allowing the cells to attach, they were exposed to serially diluted concentrations of *T. terreum* extracts (up to 1000 mg/L) for 48 and 72 h to assess cytotoxic activity. For control groups, cells were treated with equivalent concentrations of the extraction solvent. Each experiment was performed independently in duplicate, with at least four technical replicates per treatment. Cell viability was evaluated using the MTT assay, with absorbance readings taken at 570 nm using a plate reader (Thermo Scientific, Multiskan Go, Vantaa, Finland). The cell viability was calculated using formula below:

Cell viability (%) = (Absorbance of treated cells/Absorbance of control cells) × 100. Half-maximal inhibitory concentration (IC_50_) values were calculated using GraphPad Prism software (version 8.0.2). Cell cycle distribution, apoptosis assays, and gene expression analyses were performed after 72 h treatment using concentrations corresponding to the IC_50_ values and selected sub-cytotoxic doses of the extracts. The selectivity index (SI) was calculated to assess the relative cytotoxic selectivity of *T. terreum* extracts between cancerous (MCF-7) and normal (CCD-1072sk) cells. SI values were determined as the ratio of the IC_50_ value in normal cells to that in cancer cells.

### 4.6. Flow Cytometry-Based Cellular Responses

#### 4.6.1. Cell Cycle Distribution Analysis

Cell cycle progression was assessed using the Sigma-Aldrich MAK344 Cell Cycle Analysis Kit (Saint Louis, MO, USA), following the manufacturer’s instructions. MCF-7 cells were exposed to IC_50_ concentrations of the extracts for 96 h. The cells were then collected, washed with phosphate-buffered saline (PBS), and fixed in ice-cold 70% ethanol. After fixation, the cells were washed with assay buffer and incubated with a staining mixture containing RNase (enzyme A) and a DNA-binding fluorescent dye. Following incubation in the dark, the samples were analyzed using flow cytometry to determine the distribution of cells across different phases of the cell cycle. All measurements were performed in triplicate to ensure analytical reliability [67].

#### 4.6.2. Apoptosis Detection (Annexin V-FITC/PI Staining)

Apoptotic responses were evaluated using the ApopNexin™ FITC Annexin-V/PI Apoptosis Detection Kit (Merck, Darmstadt, Germany). MCF-7 cells treated with IC_50_ concentrations for 96 h were harvested, washed, and resuspended in binding buffer. They were then stained with FITC-conjugated Annexin-V and propidium iodide (PI). After a 15 min incubation at room temperature in the absence of light, the samples were analyzed by flow cytometry. This method allowed for the differentiation between viable, early apoptotic, late apoptotic, and necrotic cell populations. Cell cycle distribution was evaluated by propidium iodide (PI) staining following ethanol fixation and analyzed by flow cytometry [79].

### 4.7. Gene Expression and Metabolic Pathway Analysis

#### RT-qPCR-Based Profiling and Reactome Analysis

Gene expression profiles from MCF-7 cells treated with methanolic extracts of *T. terreum* were analyzed to understand the changes in cancer-related metabolic and signaling pathways. Differentially expressed genes were mapped to the Reactome database to identify significantly enriched biological pathways, focusing on interactions with a *p*-value of ≤0.05. Pathway enrichment analysis was conducted using a hyper-geometric test, with multiple comparisons adjusted via the Benjamini–Hochberg procedure. A false discovery rate (FDR) threshold was set at 0.05 to ensure statistical reliability. For the gene expression analysis, total RNA was isolated using a commercial silica-membrane-based kit, and RNA quality and concentration were verified using spectrophotometry. Complementary DNA was synthesized from the purified RNA, followed by quantitative real-time PCR (Analytik Jena, Jena, Germany) analysis of approximately 46 genes associated with key cancer signaling networks. Amplification reactions were conducted using SYBR Green (EuroClone, Pero, Italy) chemistry, with *GAPDH* serving as the internal reference, and relative expression levels were calculated using the 2^−ΔΔCt^ method [80]. The resulting expression patterns were integrated with the pathway enrichment results to provide mechanistic insights into the molecular responses induced by *T. terreum* treatment.

### 4.8. Statistical Analysis

The extraction experiments were conducted with three technical replicates and analyzed using JMP Pro 16 (SAS, Cary, NC, USA). Homogeneity of variance was assessed by Levene’s test, followed by One-way ANOVA. Mean comparisons were performed using Fisher’s LSD test (α = 0.05). Cell viability and IC_50_ values were determined using GraphPad Prism. Viability was calculated relative to the untreated control (100%), and IC_50_ values were obtained by nonlinear regression using a sigmoidal dose–response model. All experiments were performed with at least two independent biological replicates, each in triplicate, and results are presented as mean ± SD. Gene expression values were calculated using the 2^−ΔΔCt^ method, and flow cytometry (Beckman Coulter CytoFLEX (Brea, CA, USA)) data were analyzed using CytExpert (ver. 2.4.0.28) with a *p*-value threshold of <0.05 for statistical significance.

## 5. Conclusions

This study demonstrates that *T. terreum* possesses a unique phytochemical and volatile profile that exerts potent, selective anticancer effects on MCF-7 breast cancer cells. The most significant finding is the ethanol extract’s ability to reduce cancer cell viability to 3.64% while maintaining high safety for healthy cells. The most prominent novelty of this research is the identification of a metabolic trap mechanism, where the extract decouples glucose uptake from energy production by inhibiting key glycolytic enzymes like *ENO1* and *TPI1* while simultaneously inducing a strong oxidative stress response through *SLC7A11* upregulation. Furthermore, the molecular arrest of the cell cycle at the G0/G1 phase, driven by the severe downregulation of *DHFR* and *TK1*, provides a clear genetic basis for its antiproliferative action. Despite these promising results, the study was conducted in vitro and may not fully replicate the complex physiological environment and metabolic interactions within a living organism. Additionally, while the volatile profile was extensively characterized, the specific isolation and individual testing of high-percentage compounds like 2-acetylpyridine remain a subject for future investigation. Overall, *T. terreum* emerges as a powerful candidate for the development of metabolism-targeted, naturally derived oncological therapies. The extracts demonstrate notable biological activity and may serve as a potential source of bioactive compounds for further investigation in metabolism-targeted cancer research. However, additional studies—including protein-level validation, metabolomic analyses, testing in multiple cell models, and in vivo systems—are necessary to confirm these findings and determine their therapeutic relevance.

## Figures and Tables

**Table 1:** Total phenolic, flavonoid, and anthocyanin contents in *T. terreum* ethanol and methanol extracts.

Solvent	TPC (mg GAE/g)	TFC (mg QE/g)	Tant (mg ME/g)
Ethanol	0.65 ± 0.03 ^b^	0.62 ± 0.03 ^a^	0.61 ± 0.03 ^a^
Methanol	1.08 ± 0.04 ^a^	0.28 ± 0.01 ^b^	0.46 ± 0.02 ^b^

Means represented by different letters within the same column indicate significant differences at *p* < 0.05.

**Table 2:** Total protein and carbohydrate contents of *T. terreum* extracts depending on the solvent.

Solvent	Protein (%)	Carbohydrates (%)
Ethanol	13.15 ± 0.55 ^b^	60.06 ± 2.51 ^a^
Methanol	18.64 ± 0.78 ^a^	53.77 ± 2.25 ^a^

Means represented by different letters within the same column indicate significant differences at *p* < 0.05.

**Table 3:** Total antioxidant activities of *T. terreum* extracts from different solvents.

Solvent	DPPH (mg/g)	CUPRAC (mg/g)	ABTS (mg/g)	FRAP (mg/g)
Ethanol	0.49 ± 0.02 ^b^	1.96 ± 0.08 ^b^	1.72 ± 0.07 ^a^	3.00 ± 0.13 ^a^
Methanol	1.04 ± 0.04 ^a^	10.30 ± 0.43 ^a^	3.19 ± 0.13 ^b^	2.06 ± 0.09 ^b^

Means represented by different letters within the same column indicate significant differences at *p* < 0.05.

**Table 4:** The phenolic compounds detected in *T. terreum* by HPLC.

Phenolic and Organic Acids	Ethanol (µg/g)	Methanol (µg/g)
Gallic Acid	12.92	725.59
4-Aminobenzoic Acid	n.d.	117.16
Catechin	106.03	95.73
Chlorogenic Acid	60.80	47.01
Syringic Acid	375.09	567.90
4-Hydroxybenzoic Acid	30.57	65.50
Syringin Hydrate	160.17	120.38
Caffeic Acid	43.76	22.26
Vanillic Acid	n.d.	237.25
Ferulic Acid	63.19	101.14
Synapic Acid	n.d.	62.47
Coumaric Acid	n.d.	n.d.
Rutintrihydrate	n.d.	n.d.
Quercitrin	277.72	209.08
(−)-Epicatechin	737.95	124.56
(+)-Catechin	n.d.	n.d.
Salicylic Acid	n.d.	n.d.
Succinic Acid	134.43	247.58

n.d.: not detected.

**Table 5:** Volatile organic compound profile of *T. Terreum* characterized by SPME–GC–MS.

Group	Compound Name	%Area	RI
1. Organic Acids	Acetic acid (Ethylic acid)	43.85	716
	Hexanoic acid	1.27	1086
	Total	45.12	
2. Aldehydes	Hexanal (n-Hexanal)	1.99	818
	Benzaldehyde	1.50	1023
	Total	3.49	
3. Ketones	2-Heptanone	2.19	927
	2-Octanone	4.01	1076
	2-Decanone	0.95	1471
	Total	7.15	
4. Alcohols	2,3-Butanediol	1.81	800
	2,5,5-Trimethyl-3,6-heptadien-2-ol	0.57	1225
	4,8-Dimethyl-1-nonanol	1.24	1257
	Tridecanol	2.33	1714
	Total	5.95	
5. Lactones	4-Pentylbutan-4-olide	2.95	1795
	Total	2.95	
6. Nitrogen-Containing Heterocyclic Compounds	Methylpyrazine	0.68	843
	2,6-Dimethylpyrazine	2.07	954
	2,3-Dimethylpyrazine	0.61	963
	2,3,5-Trimethylpyrazine	1.72	1092
	2-Acetylpyridine	15.19	1144
	2-Methyl-5-trideuteromethyltetrazole	1.36	803
	Methyl carbamate	0.97	688
	Total	22.60	
7. Phenolic Compounds	Phenol, 2-methyl- (CAS) o-Cresol	1.07	1198
	Total	1.07	
8. Esters	Isoamyl phenylacetate	1.26	1571
	Total	1.26	
9. Terpenoid/Unsaturated Alcohol Derivatives	2-Undecene, 4,5-dimethyl-, (E)	1.25	1248
	Total	1.25	
	Nonane, 5-(2-methylpropyl)- (CAS) OCTANE, 4-BUTYL-2-METHYL-	0.83	1300
10. Alkanes & Alkenes	1-Nonadecene (CAS)	2.20	1696
	Heptadecane	0.64	1722
	Hexadecane	0.95	1895
	Total	4.62	
11. Aromatic/Vinyl Derivatives	Dimethylstyrene	0.84	1265
	Total	0.84	
12. Other Compounds	6-Methoxy-2-(1-buten-3-yl)-naphthalene	2.40	684
	1,4-Epoxycyclohex-2-ene	1.30	1050
	Total	3.70	

## Data Availability

The original contributions presented in this study are included in the article. Further inquiries can be directed to the corresponding author.

## Abbreviations

The following abbreviations are used in this manuscript:

*T. terreum*	*Tricholoma terreum*
CUPRAC	Cupric reducing antioxidant capacity
ABTS	2,2′-azino-bis(3ethylbenzothiazoline-6-sulfonic acid
DPPH	2,2-diphenyl-1-picrylhydrazyl
MTT	[3-(4,5-dimethylthiazol-2-yl)-2,5-diphenyltetrazolium bromide]
PCR	Polymerase chain reaction
DMEM	Dulbecco’s modified eagle medium
EDTA	Ethylenediaminetetraacetic acid
DMSO	Dimethyl sulfoxide
PPARalpha	Peroxisome proliferator-activated receptor alpha
TPC	Total phenolic content
TFC	Total flavonoid content
VOCs	Volatile organic compounds
